# Prognostication of responsive neurostimulation system responsiveness using presurgical magnetoencephalography

**DOI:** 10.1093/braincomms/fcac114

**Published:** 2022-05-09

**Authors:** Rasesh B. Joshi, Hitten P. Zaveri

**Affiliations:** 1 Department of Neurology, Boston Children’s Hospital, Harvard Medical School, Boston, MA, USA; 2 Department of Neurology, Yale University, 333 Cedar Street, New Haven, CT 06520, USA

## Abstract

This scientific commentary refers to ‘Network connectivity predicts effectiveness of responsive neurostimulation in focal epilepsy’, by Fan *et al.* (https://doi.org/10.1093/braincomms/fcac104)

**This scientific commentary refers to ‘Network connectivity predicts effectiveness of responsive neurostimulation in focal epilepsy’, by Fan *et al.* (https://doi.org/10.1093/braincomms/fcac104)**

Epilepsy is one of the most common neurological disorders, affecting up to 1% of the world population. Although most patients with epilepsy can achieve adequate seizure control with anti-seizure medications, approximately one-third of patients with epilepsy will continue to have seizures despite multiple trials of various medication regimens and are considered medically refractory.^1^ In patients with focal epilepsies, in whom seizures are thought to emanate from spatially restricted pathologic tissue, resection of this seizure onset zone (SOZ) can be a viable treatment option. Neurosurgical outcomes vary significantly by SOZ localization and type of approach, and one meta-analysis reports anywhere from 41 to 79% of patients remain seizure-free for 5–10 years post-surgery.^2^ A subset of patients, however, may continue to experience seizures, sometimes from seizure foci that were not identified in earlier evaluations. A number of observations from human and animal studies, including observations on surgical outcome, have increasingly supported the network theory of epilepsy which holds that even in presumed focal epilepsies, seizures arise from aberrant networks^3^ and ictogenesis involves both hyperexcitability and cascading, pathologic synchrony.

Indeed, multiple studies suggest that network nodes around the clinically defined SOZ and spatially distant from it may be equally important for the onset and evolution of a seizure as those within the SOZ.^4^ Taking this idea further, a fruitful avenue of study has been in the use of functional connectivity (FC) measures to identify differences in epilepsy networks in patients who responded well to epilepsy surgery and those who did not. Interestingly, many of these studies have shown differences in network structure in the SOZ and elsewhere in resting interictal data, suggesting that the epilepsy network is persistently abnormal between seizures and network aberrance can be detected independently of seizure.^5^ Of particular interest in network-driven treatment modalities for epilepsy is the role of device-based therapies, particularly in patients who may have multiple, distributed seizure foci or in whom resection is not feasible. The responsive neurostimulation (RNS®) System has shown promising results in reducing seizure frequency at a population level, but individual response can show a high degree of variability.^6^ To date, no reliable measures can distinguish those who would be expected to respond well versus those who would not, much like the difficulty in predicting outcomes for traditional resective surgeries. In their recent article in Brain Communications, Fan *et al*.^7^ provide a fascinating and well-constructed analysis of resting-state magnetoencephalography (MEG) data collected from patients who went on to have RNS device implantations and show that FC measures could provide a patient-specific biomarker for providing an *a priori* expected RNS responsiveness pre-implantation (Fig. 1).

**Figure 1:**
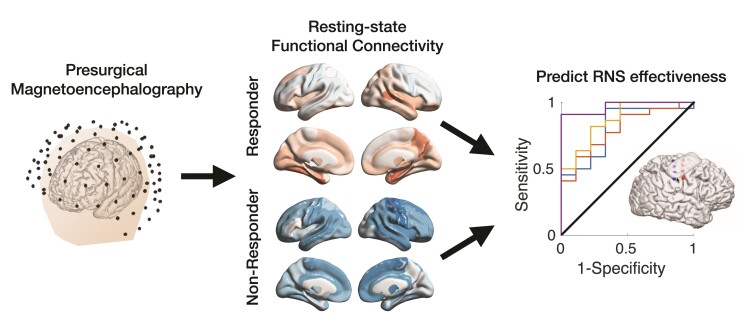
The main findings of Fan *et al*.^7^ are summarized in this figure. Thirty-one patients underwent MEG (*left*) before implantation of a RNS device. Resting-state FC was computed and normalized to healthy individuals. Example FC maps in the alpha frequency range are shown (*middle*). Regional and global FC measures were used to predict the effectiveness of RNS therapy (*right*). Responders to RNS exhibited increased FC compared to non-responders. Figure courtesy of study authors.

To accomplish this, the authors studied MEG recordings from a cohort of patients who underwent routine EEG (electroencephalography)/MEG and were subsequently implanted with the RNS System over a 6-year period at a single medical centre. Resting MEG data were retrospectively analysed for each patient from brief artifact-free recordings, and importantly, patients were on their normal doses of anti-seizure medications. Imaginary coherence, a phase-based measure of connectivity that is not influenced by volume conduction,^8^ was used to compute FC maps of study patients normalized to those of healthy control subjects. These measures were obtained for traditional EEG/MEG frequency bands and the cortical parcellations of the Brainnetome atlas.^9^ FC was subsequently computed for global and regional (hemispheric and lobar) representations. Post-implantation outcomes were determined from patient-reported seizure frequency and divided into those with ≥50% reduction in seizures (responders) versus those with <50% reduction. After examining differences in FC between responders and non-responders, the authors used this information to construct binary outcome logistic regression models for classifying patient outcomes.

In order to minimize the risk of confounding variables, the authors rigorously assessed a variety of factors including patient information, seizure characteristics, medications, RNS configuration, and others and found no significant difference between responders and non-responders. The healthy control group was age and gender matched to the study population. Interestingly, the results showed that increased FC was associated with favourable outcomes to surgery, whereas decreased FC was associated with poor response. Specifically, global FC in the alpha and beta bands showed a distinct difference between two groups and was used in classifying patients with reasonable accuracy. Of particular interest for the purpose of this commentary was the fact that global FC was a stronger predictor than regional measures, and the specificity in frequency band in the result. The authors speculate that a plausible explanation for this observation could be that epilepsy networks that are more globally coherent may be more ‘permissive’ to stimulation and entrainment through the RNS System, and thus easier to control.

This study is particularly exciting as it provides one of the first examinations of neurophysiological underpinnings of individual patient responses to RNS System implantation in a manner that allows prognostication of RNS System responsiveness. The results observed, particularly the spatial and spectral aspects, warrant further exploration and could open further avenues for work in achieving optimal control of network-level aberrance in medically refractory epilepsy. From a clinical standpoint, this is also promising as it is a step towards a method for non-invasively predicting whether patients could be expected to respond well to RNS, without having to go through the full process of surgical evaluation (i.e. intracranial electrode implantation, monitoring, medication taper) and the risks associated with it.

As noted in the study, one significant limitation is the number of patients considered, which may eventually affect generalization of the results. A relatively small number of patients make the results more susceptible to subject heterogeneity. This applies not just to patient characteristics, but also to factors such as self-reporting of seizures and assessment of seizure reduction. It is to the study’s credit, however, that a large number of potential confounds were accounted for, and errors in seizure reporting were mitigated by incorporating data from multiple clinical assessments.

Importantly, this study raises exciting new questions and opens the door to several potential avenues for research. First, it would be valuable to replicate these results in a large-cohort, prospective study examining FC measures in patients undergoing RNS System implantation. Second, it raises questions about the mechanistic underpinnings of oscillatory dynamics in epilepsy networks and the role they play in seizure generation, propagation and termination, particularly in the role of alpha and beta bands and global, modulatory phenomena in the brain. Third, the study raises intriguing questions about stimulation therapy. RNS is designed to deliver targeted therapy that is spatiotemporally specific. Previous indications, however, have been that temporal specificity is not central to the efficacy of RNS (i.e. stimulation can be delivered for all event detections), and this study could raise the possibility that strict spatial targeting may not be central either. It remains to be determined if the band-limited increase in global FC in responders suggests that stimulation therapy should be optimized in some manner to increase global FC or activity in the alpha or beta bands. Finally, MEG is a promising platform for future study with its non-invasive whole-brain coverage and strong temporal resolution. Through various source reconstruction methods, it also may provide a window into deep, subcortical activity which cannot be interrogated with non-invasive EEG.^10^ To this end, further and more granular exploration of the role of subcortical structures in epilepsy networks and predicting neurosurgical response may be fruitful.

## Data Availability

Data sharing is not applicable to this article as no new data were created or analysed.
